# Transient and Persistent UP States during Slow-wave Oscillation and their Implications for Cell-Assembly Dynamics

**DOI:** 10.1038/s41598-018-28973-y

**Published:** 2018-07-16

**Authors:** Chi Chung Alan Fung, Tomoki Fukai

**Affiliations:** RIKEN Center for Brain Science, Hirosawa 2-1, Wako City, Saitama 351-0198 Japan

## Abstract

The membrane potentials of cortical neurons *in vivo* exhibit spontaneous fluctuations between a depolarized UP state and a resting DOWN state during the slow-wave sleeps or in the resting states. This oscillatory activity is believed to engage in memory consolidation although the underlying mechanisms remain unknown. Recently, it has been shown that UP-DOWN state transitions exhibit significantly different temporal profiles in different cortical regions, presumably reflecting differences in the underlying network structure. Here, we studied in computational models whether and how the connection configurations of cortical circuits determine the macroscopic network behavior during the slow-wave oscillation. Inspired by cortical neurobiology, we modeled three types of synaptic weight distributions, namely, log-normal, sparse log-normal and sparse Gaussian. Both analytic and numerical results suggest that a larger variance of weight distribution results in a larger chance of having significantly prolonged UP states. However, the different weight distributions only produce similar macroscopic behavior. We further confirmed that prolonged UP states enrich the variety of cell assemblies activated during these states. Our results suggest the role of persistent UP states for the prolonged repetition of a selected set of cell assemblies during memory consolidation.

## Introduction

Various types of oscillations appear in neural systems depending on the state of the animal^1^. During non-rapid-eye-movement (NREM) sleep^2–4^ or in anesthetized states^5^, the electroencephalogram (EEG) signals recorded from the neocortex exhibit slow-wave oscillation (1 H*z*), in which cortical neurons show spontaneous bistable fluctuations of the membrane potentials between UP state and DOWN state. Slow-wave oscillation is widely believed to play a crucial role in memory consolidation^6^ and its impairment is known to cause mental disorders^7^.

Many attempts have been made to clarify the underlying mechanisms of the UP-DOWN transitions^8^. It is thought that the transitions are regulated by thalamocortical input to the cortex^9,10^. However, while the removal of thalamus significantly suppresses the occurrence of UP states, this manipulation does not completely eliminate the bistable transitions, suggesting that local cortical networks have a capability for generating the UP state^11^. Therefore, the UP-DOWN transitions are generated and maintained by a state-dependent interplay between cortical network dynamics and thalamocortical inputs.

There are numerous theoretical studies on the possible neural mechanisms of UP-DOWN transition, including networks of rate neuron model and spiking neuron models. A biologically realistic thalamocortical network model was proposed to describe slow-wave oscillation and transitions to a continuous UP state corresponding to awake state of animals^12^. A neural network model was constructed to replicate the behavior of single cells and their network during slow-wave oscillation in control and under pharmacological manipulations^13^. The roles of intrinsic properties such as low-threshold bursting and spike-frequency adaptation in the generation of spontaneous cortical activities were also explored^14^. Sustained UP-DOWN transitions were shown to self-organize through spike-time-dependent plasticity in a recurrent network model of excitatory and inhibitory neurons^15^. Clustered synaptic connections were studied as a possible source of highly variable temporal patterns in the slow cortical dynamics^16^. The mechanisms of highly variable UP-DOWN transitions were experimentally and computationally explored to reveal various dynamical regimes in a bistable network driven by fluctuating input^17^.

It has recently been shown that the temporal patterns of the UP-DOWN transition are qualitatively different between the layer 3 of the medial entorhinal cortex (MECIII) and the layer 3 of the medial entorhinal cortex (LECIII) in an interesting way^4^. While the temporal patterns are synchronized in LECIII with those of UP-DOWN transitions in neocortical areas, UP states often continue in MECIII during several cycles of neocortical UP-DOWN transitions. Though differences in the underlying circuit structure were suggested to underlie the distinct activity patterns between MECIII and LECIII, the cause of persistent UP state remains unknown. These findings motivated us to explore whether and how distinct network configurations modulate the temporal patterns of the UP-DOWN transition. Furthermore, what do the various UP-DOWN transition patterns imply for the role of slow-wave oscillation in memory processing?

We study these questions in networks of spiking neurons^18^ randomly connected with three distinct types of synaptic weight distributions. In each network, the weights of connections between neurons are random numbers drawn from pre-defined distributions with given mean and variance. We first conduct a mean-field analysis to clarify the role of excitatory-inhibitory strength ratio (E-I ratio) in the occurrence and sustainability of UP states. The results are further verified by numerical simulations. In all the network types, we found that the mean and variance of connection weights are critical to determine the macroscopic network behavior. Then, we numerically explore the statistical features of UP-DOWN state transitions in different parameter settings and compare the results with the experimental observations reported in the literature^4^. Furthermore, to investigate the computational implications of slow oscillation for memory consolidation, we analyze the variability of neural ensemble activity patterns during UP states in all the networks. We demonstrate that the variety of activated neuron ensembles reduces rather than increases with time passage from the onset of an UP state, implying that the networks repeat to activate a set of selective neuron ensembles during memory consolidation.

## Model and Methods

We use randomly-connected-recurrent networks of spiking neurons. Our spiking neuron model is based on the adaptive exponential integrate-and-fire model (AdEx)^18^. For simplicity, excitatory neurons and inhibitory neurons are modeled by identical spiking neuron models. Synaptic weights are drawn from the different distributions described below, and all synaptic connections are randomly wired. Because the neuron model does not have intrinsic bi-stability, the UP-DOWN transitions obtained in this study represent pure effects of network dynamics with given network configuration.

### Single Neuron Model

Dynamics of the membrane potential of each neuron is given by

1 $$\begin{array}{rcl}C\frac{d{V}_{{\rm{X}}}(t)}{dt} & = & -\,{g}_{{\rm{L}}}({V}_{{\rm{X}}}(t)-{V}_{{\rm{L}}})+\mathop{\underbrace{{g}_{{\rm{L}}}{{\rm{\Delta }}}_{{\rm{T}}}\,\exp (\frac{{V}_{{\rm{X}}}(t)-{V}_{{\rm{T}}}}{{{\rm{\Delta }}}_{T}})}}\limits_{\ast }\mathop{\underbrace{\{1-\exp [-{(\frac{{[t-{t}^{{\rm{sp}}}]}_{+}}{2{\rm{m}}s})}^{20}]\}}}\limits_{\#}\\  &  & -{u}_{{\rm{X}}}(t)+{I}_{{\rm{syn}}}^{{\rm{X}}}(t)+{I}_{{\rm{input}}}^{{\rm{X}}}(t)+\eta (t),\end{array}$$

where X ∈ {E, I} labels excitatory or inhibitory neuron. *C* is the membrane capacitance and *g*_L_ is the conductance of leaky current. The term labeled with asterisk is responsible for spike generation, while the term labeled with sharp mark induces a refractory period of 2 ms. Time *t*^sp^ is the time of the latest spike, *V*_T_ the effective threshold for spike generation, and Δ_T_ the width of the range of membrane potential for spike generation. Equation (1) diverges during spike generation. The moment when the membrane potential reaches to *V*_X_(*t*) = 20 m*V* is regarded as the time of spiking. The moment of the *i*-th spike is denoted by ${t}_{i}^{{\rm{sp}}}$. At $t={t}_{i}^{{\rm{sp}}}$, *V*_X_(*t*) is reset to −55 mV. The variable *u*_X_(*t*) describes spike-frequency adaptation (SFA) according to^18^

2 $${\tau }_{u}\frac{d{u}_{{\rm{X}}}(t)}{dt}=-\,{u}_{{\rm{X}}}(t)+{a}_{u}({V}_{{\rm{X}}}(t)-{V}_{{\rm{L}}})+{b}_{u}\delta (t-{t}^{{\rm{sp}}}),$$

where *a*_*u*_ is the parameter controlling the adaptation induced by depolarization and *b*_*u*_ is the adaptation current induced by spikes. The parameters used in the present simulations are as follows: *C* = 150 pF, *g*_L_ = 10.005 nS, *V*_L_ = −70 mV, Δ_T_ = 2 mV, *V*_T_ = −55 mV, *τ*_*u*_ = 200 ms, *a*_*u*_ = 4.0 nS and *b*_*u*_ = 50.0 pA. ${I}_{{\rm{syn}}}^{{\rm{X}}}(t)$ is the current evoked by synaptic input from pre-synaptic neurons, and ${I}_{{\rm{input}}}^{{\rm{X}}}(t)$ is an external input to the network, which is given by a Poisson spike train. The details of the synaptic currents are given below.

### Synaptic Couplings

The network consists of two groups of neurons: excitatory neurons and inhibitory neurons. For simplicity, we model both neuron types by Eqs (1) and (2), with X = E referring to excitatory neurons and X = I to inhibitory neurons. Inhibitory neurons do not receive external input, i.e., ${I}_{{\rm{input}}}^{{\rm{I}}}=0$. Neurons from both groups are interconnected by multiple types of synapses, which are AMPA-, NMDA-, fast GABA-A-, slow GABA-A- and post-synaptic GABA-B-receptor mediated synapses.

In this study, all synapses obey second-order kinetics, with *s*_*ψ*,*j*_ being the gating functions of synapse type *ψ*, where *ψ* denotes the type of synapses mentioned in the previous paragraph. The dynamics of synapses responding to spikes of pre-synaptic neuron *j* are given by

3 $$\frac{d{x}_{\psi ,j}}{dt}=(\frac{1}{{\tau }_{\psi ,{\rm{up}}}})\sum _{{t}_{j}^{{\rm{sp}}} < t}\delta (t-{\tau }_{{\rm{axon}}}-{t}_{j}^{{\rm{sp}}})-\frac{{x}_{\psi ,j}}{{\tau }_{\psi ,{\rm{up}}}}$$

4 $$\frac{d{s}_{\psi ,j}}{dt}={x}_{\psi ,j}(1-{s}_{\psi ,j})-\frac{{s}_{\psi ,j}}{{\tau }_{\psi ,{\rm{dn}}}}.$$

where *τ*_axon_ = 1 ms is the axonal delay of pre-synaptic neuron. Rising time constants are set as *τ*_AMPA,up_ = 0.5 ms^19^, *τ*_NMDA,up_ = 5.8 ms^20^, *τ*_GABA−A−fast,up_ = 1.8 ms^21^, *τ*_GABA−A−slow,up_ = 1.8 ms^21^ and *τ*_GABA−B−post,up_ = 100 ms^22^. Decay time constants are *τ*_AMPA,dn_ = 4.0 ms^19^, *τ*_NMDA,dn_ = 87.5 ms^20^, *τ*_GABA−A−fast,dn_ = 12.0 ms^21^, *τ*_GABA−A−slow,dn_ = 47.0 ms^21^ and *τ*_GABA−B−post,dn_ = 500 ms^22^.

For an excitatory neuron, the synaptic current, ${I}_{{\rm{syn}}}^{{\rm{E}}}$, is given by

5 $${I}_{{\rm{syn}}}^{{\rm{E}}}={\gamma }_{{\rm{E}}}\sum _{\psi ,j}{J}_{\psi ,j}^{{\rm{EE}}}{g}_{\psi }{s}_{\psi ,j}(t)({V}_{{\rm{R}},\psi }-{V}_{{\rm{E}}}(t))+{\gamma }_{{\rm{I}}}\sum _{\psi ,j}{J}_{\psi ,j}^{{\rm{EI}}}{g}_{\psi }{s}_{\psi ,j}(t)({V}_{{\rm{R}},\psi }-{V}_{{\rm{E}}}(t)),$$

and the synaptic current of inhibitory neurons, ${I}_{{\rm{syn}}}^{{\rm{I}}}$, is given by

6 $${I}_{{\rm{syn}}}^{{\rm{I}}}={\gamma }_{{\rm{E}}}\sum _{\psi ,j}{J}_{\psi ,j}^{{\rm{IE}}}{g}_{\psi }{s}_{\psi ,j}(t)({V}_{{\rm{R}},\psi }-{V}_{{\rm{E}}}(t))+{\gamma }_{{\rm{I}}}\sum _{\psi ,j}{J}_{\psi ,j}^{{\rm{II}}}{g}_{\psi }{s}_{\psi ,j}(t)({V}_{{\rm{R}},\psi }-{V}_{{\rm{E}}}(t))\mathrm{.}$$

Here, the reversal potentials *V*_R,*ψ*_ are set as follows: *V*_R,*AMPA*_ = 0 mV^15^, *V*_R,NMDA_ = 0 mV^15^, *V*_R,GABA−A_ = −70 mV^23^, *V*_R,GABA−B_ = −80 mV^24^. The conductances *g*_*ψ*_ are set as follows: *g*_AMPA_ = 1.05 nS, *g*_NMDA_ = 1.05 nS, *g*_GABA−A_ = 4.0 nS and *g*_GABA−B_ = 2.0 nS. For the conductance, only ratios between different kinds of receptors are important, because effective magnitudes in simulations will be controlled by scaling factors. The ratios are chosen so that DOWN state could be comparable to the DOWN-state membrane potentials shown in experiments^25^. The parameters *γ*_E_ and *γ*_I_ in Eqs (5) and (6), termed excitatory or inhibitory weight scaling factor, are to be varied in simulations for different scenarios. In real systems, neurons should be influenced by more classes of synapses. Here we consider only four classes of synapses to represent fast-excitatory, slow-excitatory, fast-inhibitory and slow-inhibitory connections. Also, one should note that *γ*_E_/*γ*_I_ can be interpreted as the excitatory-inhibitory ratio for physical connections, e.g. number of synapses and destiny of spines. It should not be confused with the postsynaptic current ratio reported by Beed *et al*.^26^.

**Figure 1:**
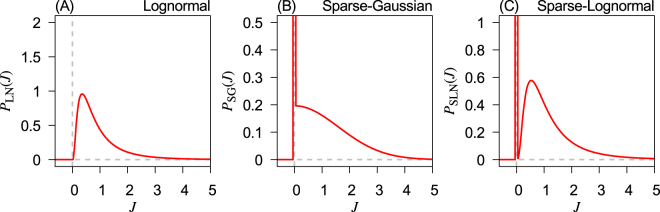
Probability density functions used to generate random connection strengths between neurons. (**A**) The probability density function for simple log-normal random numbers presented in Eq. (7). (**B**) The probability density function for sparse-Gaussian random numbers presented in Eq. (8). (**C**) The probability density function for sparse-log-normal random numbers presented in Eq. (9). Parameter: *σ* = 1.0.

In this study, the strength of connections between neurons, ${J}_{\psi }^{{\rm{EE}}}$, ${J}_{\psi }^{{\rm{EI}}}$, ${J}_{\psi }^{{\rm{IE}}}$ and ${J}_{\psi }^{{\rm{II}}}$, obeys one of the following statistical distributions: (1) log-normal distribution, (2) sparse-Gaussian distribution and (3) sparse-log-normal distribution. A log-normal distribution has a long tail and can mathematically result from the product of independent random numbers. In this study, we are interested in examining the dynamical properties of neural network that depend (or do not depend) on the statistical details of random connection weights. There is evidence that spine sizes or synaptic weights of excitatory synapses as well as other properties of cortical circuits are log-normally distributed^27^. Some report shows the strength of inhibitory synapses also obeys a long-tailed distribution^28^. Thus, we use the log-normal probability density function given by

7 $$\begin{array}{rcl}{P}_{\mathrm{LN}}({J}_{\psi }^{{\rm{XX}}^{\prime} }) & = & \frac{1}{{J}_{\psi }^{{\rm{XX}}^{\prime} }{\sigma }_{\mathrm{LN}}^{{\rm{XX}}^{\prime} }\sqrt{2\pi }}\exp \,[-\frac{{(\mathrm{ln}{J}_{\psi }^{{\rm{XX}}^{\prime} }-{\mu }_{\mathrm{LN}}^{{\rm{XX}}^{\prime} })}^{2}}{2{\sigma }_{\mathrm{LN}}^{{\rm{X}}X^{\prime} 2}}]\\ {\sigma }_{\mathrm{LN}}^{{\rm{XX}}^{\prime} 2} & = & \mathrm{ln}({\sigma }^{2}+1)\\ {\mu }_{\mathrm{LN}}^{{\rm{XX}}^{\prime} } & = & -\,\frac{1}{2}\,\mathrm{ln}({\sigma }^{2}+1),\end{array}$$

where X, X′ ∈ {E, I}, and parameters ${\mu }_{\mathrm{LN}}^{{\rm{XX}}^{\prime} }$ and ${\sigma }_{\mathrm{LN}}^{{\rm{XX}}^{\prime} }$ are determined by the variance *σ*^2^ of the distribution with a fixed mean 1.0. Figure 1(A) shows an example for *σ*^2^ = 1.0. The probability density function of the sparse-Gaussian distribution is a sum of a delta function and a truncated Gaussian function peaked at 0:

8 $$\begin{array}{rcl}{P}_{{\rm{SG}}}({J}_{\psi }^{{\rm{XX}}^{\prime} }) & = & (1-a)\delta ({J}_{\psi }^{{\rm{XX}}^{\prime} })+a\times \frac{1}{{\sigma }_{{\rm{SG}}}^{{\rm{XX}}^{\prime} }\sqrt{2\pi }}{\rm{e}}{\rm{x}}{\rm{p}}(-\frac{{J}_{\psi }^{{\rm{XX}}^{\prime} 2}}{2{\sigma }_{{\rm{SG}}}^{{\rm{XX}}^{\prime} 2}}){\rm{\Theta }}({J}_{\psi }^{{\rm{XX}}^{\prime} })\\ {\sigma }_{{\rm{SG}}}^{{\rm{XX}}^{\prime} } & = & \sqrt{\frac{2}{\pi }}({\sigma }^{2}+1)\\ a & = & \frac{\pi }{2}\frac{1}{({\sigma }^{2}+1)},\end{array}$$

where Θ is the Heaviside step function, *a* is the sparseness and ${\sigma }_{{\rm{SG}}}^{{\rm{XX}}^{\prime} }$ the width of the truncated Gaussian function. One example for the probability density function is shown in Fig. 1(B). Since the mean and the variance of the truncated Gaussian distribution are coupled, the inclusion of the sparseness parameter *a* enabled us to control the mean and the variance of those random numbers sampled from the distribution independently. The sparse-log-normal is defined in a similar manner:

9 $$\begin{array}{rcl}{P}_{{\rm{SLN}}}({J}_{\psi }^{{\rm{XX}}^{\prime} }) & = & (1-a)\delta ({J}_{\psi }^{{\rm{XX}}^{\prime} })+a\times \frac{1}{{J}_{\psi }^{{\rm{XX}}^{\prime} }{\sigma }_{{\rm{SLN}}}^{{\rm{XX}}^{\prime} }\sqrt{2\pi }}\exp \,[-\frac{{(\mathrm{ln}{J}_{\psi }^{{\rm{XX}}^{\prime} }-{\mu }_{{\rm{SLN}}}^{{\rm{XX}}^{\prime} })}^{2}}{2{\sigma }_{{\rm{SLN}}}^{{\rm{XX}}^{\prime} 2}}]\\ {\sigma }_{{\rm{SLN}}}^{{\rm{XX}}^{\prime} 2} & = & {\rm{ln}}\,[a({\sigma }_{{\rm{e}}}^{2}+1)]\\ {\mu }_{{\rm{SLN}}}^{{\rm{XX}}^{\prime} } & = & {\rm{l}}{\rm{n}}(\frac{1}{a})-\frac{1}{2}\,\mathrm{ln}({\sigma }^{2}+1)\\ a & = & \frac{\pi }{2}\frac{1}{{\sigma }^{2}+1},\end{array}$$

where *a* is the sparseness, ${\sigma }_{{\rm{SLN}}}^{{\rm{XX}}^{\prime} }$ and ${\mu }_{{\rm{SLN}}}^{{\rm{XX}}^{\prime} }$ are parameters for the log-normal components. There is one example shown in Fig. 1(C) with *σ*^2^ = 1.0. These two distributions also have mean 1.0 and variance *σ*^2^. The sparseness parameter *a* has also been included in sparse-log-normal random number for comparison with sparse-Gaussian random connection weights.

In order to make fair comparison, the means of all the probability density functions were fixed at 1.0. For I-to-E and I-to-I connections (i.e., XX′ ∈ {EI, II}), the variances were fixed at 1.0, while E-to-E and E-to-I connections (XX′ ∈ {EE, IE}) had variance ${\sigma }_{{\rm{E}}}^{2}$. In summary, the tunable parameters are *γ*_E_, *γ*_I_ and ${\sigma }_{{\rm{E}}}^{2}$, where *γ*_E_ and *γ*_I_ scale excitatory connections and inhibitory connections, and ${\sigma }_{{\rm{E}}}^{2}$ controls the variance of excitatory connections. With this setting, the inputs to excitatory neurons and inhibitory neurons will be statistically similar, which seems to be unrealistic. However, the sustainable activity is mainly supported by E-E connections, while the adjustment for contributions from inhibitory neurons can be done by controlling *γ*_I_. Also, since the inhibitory connection has a smaller variance (*σ*^2^ = 1.0), configuration detail of the excitatory input towards inhibitory neurons should not make a significant difference to the inhibitory feedback. In addition, this setting enabled us to simplify the analysis of the model using the mean-field analysis to search for condition for UP-state occurrence. The current setting was chosen to make the model simple enough to analyze without lose of qualitative details.

### Numerical Simulations

In most simulations, there are *N*_E_ = 1000 excitatory neurons and *N*_I_ = *N*_E_/4 inhibitory neurons. The radio between excitatory neurons and inhibitory neurons is chosen based on the data in a report by Mizuseki *et al*.^29^. In which, the ratio between excitatory neurons and inhibitory neurons in the entorhinal cortex is 3.9 ± 0.7. The differential equations (1), (2), (3) and (4) were integrated using second-order Runge-Kutta methods. In numerical simulations of neural population, all measurements were made after a 2-second transient period to initialize the dynamical variables. For further verifications of size effects, *N*_E_ = 2000 was also used in some simulations. The results for *N*_E_ = 2000 are presented in online supplementary information.

## Results

We constructed three neural network models with different distributions (log-normal, sparse-log-normal and sparse Gaussian) of synaptic weights and investigated the statistical properties of UP-DOWN state transitions generated by these models. Below, we show both analytical and numerical results. First, the basic properties of single neurons are studied numerically and analytically. Then, we demonstrate the statistical properties of neural networks with random connection weights obeying different distributions. Finally, we explore the ability of these networks in generating synchronous activity patterns of cell assemblies during UP states.

### Single Neuron Behvaior

**Figure 2:**
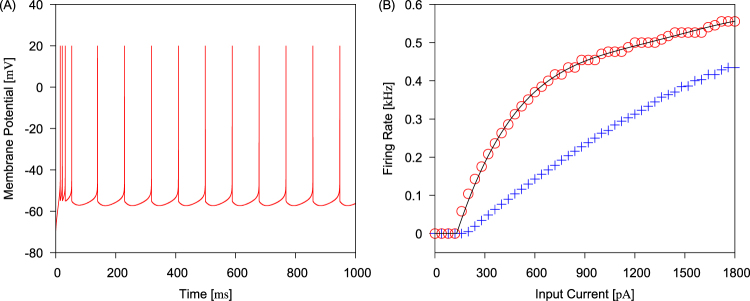
Response of a single neuron modeled by Eq. (1). (**A**) Traces of the membrane potential response evoked by a steady input current are shown. Parameter values were set as *a*_*u*_ = 4.0 nS, *b*_*u*_ = 50 mA and ${I}_{{\rm{input}}}^{{\rm{X}}}=300\,{\rm{mA}}$. (**B**) Firing rate of a neuron versus input current ${I}_{{\rm{input}}}^{{\rm{X}}}$ is plotted. Circles show results of simulations without SFA and solid line shows a fit by the polynomial given in Eq. (10). Crosses show results of simulations with SFA.

For a later use in the mean-field analysis of network dynamics, we derive the response curve of our neuron model. In the following simulations, the synaptic input term ${I}_{{\rm{syn}}}^{{\rm{X}}}$ was dropped from Eq. (1), while ${I}_{{\rm{input}}}^{{\rm{X}}}$ was fixed at a constant current 300 pA. A typical example of the membrane dynamics during sustained firing is shown in Fig. 2(A) for *a*_*u*_ = 4.0 nS and *b*_*u*_ = 50 pA. The instantaneous firing rate declines to a steady value due to SFA.

The firing rate of a single neuron without SFA (*a*_*u*_ = 0 nS and *b*_*u*_ = 0 mA) is plotted in Fig. 2(B) as a function of input current, which shows a non-linear property. The firing rate increases continuously for a steady input current larger than the input current threshold *θ*_*f*_ ≈ 130 pA, implying that the neuron model has the type-I excitability as in cortical pyramidal neurons^30^. The firing rate saturates as the input current increases. With SFA, the firing rate is suppressed, as shown in Fig. 2(B), and increases approximately in proportion to the strength of input current. The nonlinear input-output curve of the neuron without SFA is fitted as *F*(*I*) = *f*(*I* − *θ*_*f*_) by means of the following polynomial:

10 $$f(I)={\rm{\Theta }}(I)[AI+B{I}^{2}+C{I}^{3}+D{I}^{4}],$$

where the coefficients *θ*_*f*_ ≈ 130 pA, *A* = 1.32 Hz/pA, *B* = −1.47 × 10^−3^ Hz/(pA)^2^, *C* = 7.86 × 10^−7^ Hz/(pA)^3^ and *D* = −1.56 × 10^−10^ Hz/(pA)^4^ for ${I}_{{\rm{input}}}^{{\rm{X}}}-{\theta }_{f} < 1840\,{\rm{pA}}$. Θ is the Heaviside step function. For larger values of input current, neuronal activity is almost saturated. The response function will be used as the firing rate of a neuron responding to input current *I* in the mean-field analysis of networks of the model neurons.

### UP-DOWN Transitions in Network Simulations

Next, we explore the dynamics of recurrent networks consisting of the neuron models receiving afferent input, which is described by a stationary Poisson spike train of rate 10 Hz:

11 $${I}_{{\rm{input}}}^{{\rm{X}}}(t)={\gamma }_{{\rm{input}}}{J}^{{\rm{input}}}{s}_{{\rm{input}}}(t)$$

12 $$\frac{d{x}_{{\rm{input}}}}{dt}=(\frac{1}{{\tau }_{{\rm{input}},{\rm{up}}}})\sum _{{t}_{{\rm{input}}}^{{\rm{sp}}} < t}\delta (t-{\tau }_{{\rm{axon}}}-{t}_{{\rm{input}}}^{{\rm{sp}}})-\frac{{x}_{{\rm{input}}}}{{\tau }_{{\rm{input}},{\rm{up}}}}$$

13 $$\frac{d{s}_{{\rm{input}}}}{dt}={x}_{{\rm{input}}}(1-{s}_{{\rm{input}}})-\frac{{s}_{{\rm{input}}}}{{\tau }_{{\rm{input}},{\rm{dn}}}},$$

where *γ*_input_ is a scaling factor, *γ*_input_ = *γ*_E_, *J*^input^ is the weight of afferent synapse, which obeys the same distribution as recurrent connections (log-normal, sparse-Gaussian or sparse-log-normal) with ${\sigma }_{{\rm{input}}}^{2}=1.0$, *s*_input_(*t*) is the gating function with the raising time *τ*_input,up_ = 1 m*s* and *τ*_input,dn_ = 20 m*s*, and ${t}_{{\rm{input}}}^{{\rm{sp}}}$ is input spike time.

**Figure 3:**
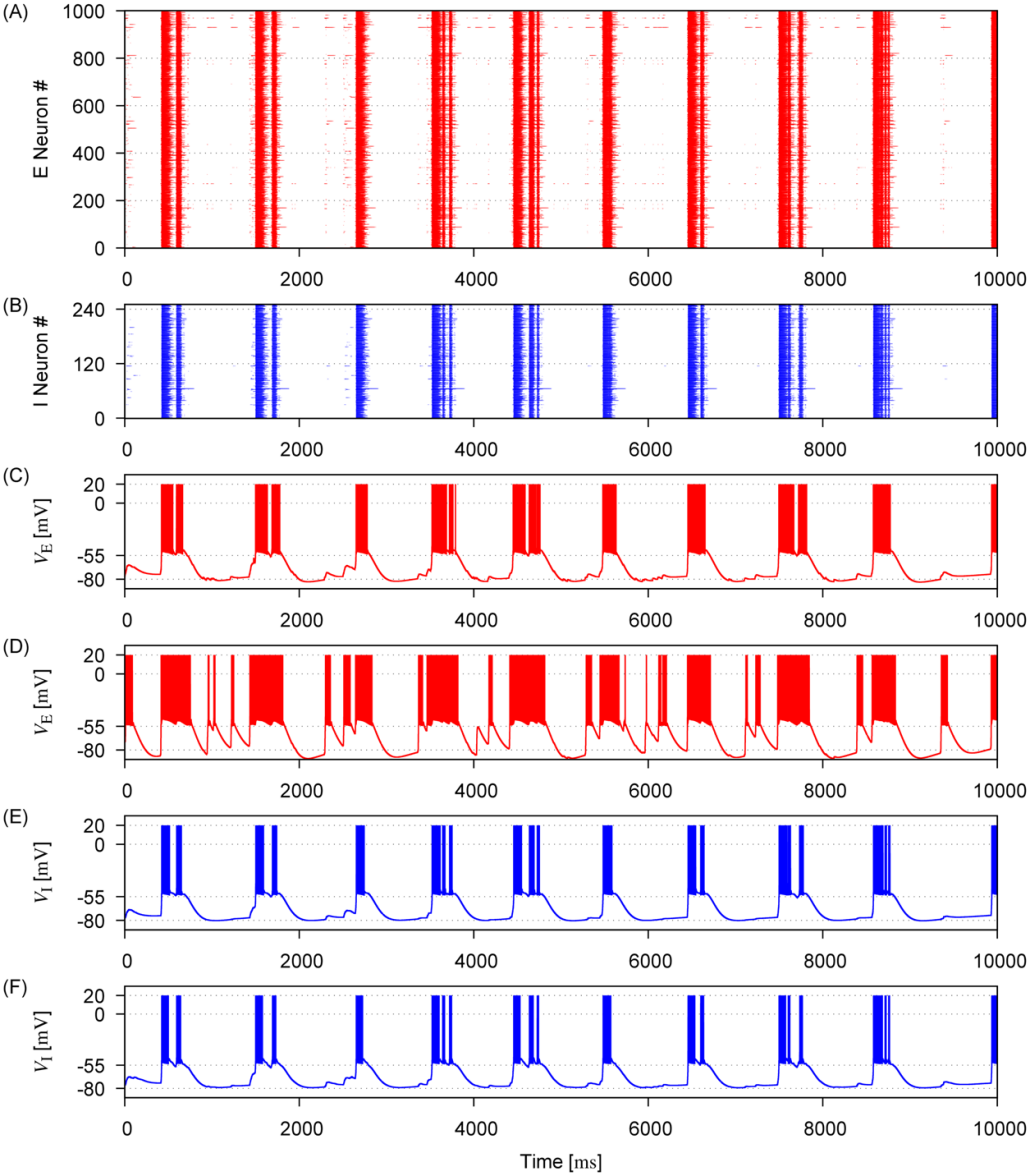
Network activity obtained for a log-normal weight distribution. (**A**) Spike trains of excitatory neurons demonstrate synchronous activity of these neurons. (**B**) Spike trains of inhibitory neurons are shown. (**C**,**D**) Membrane potentials of two excitatory neurons are displayed as examples. (**E**,**F**) Membrane potentials are shown for two inhibitory neurons. Parameter values are set as *γ*_E_ = 0.1 and *σ*_E_ = 10.

Figure 3(A,B) shows spike raster of excitatory and inhibitory neurons during synchronous UP-DOWN transitions in a recurrent network with log-normal connection weights, where parameter values are *γ*_E_ = *γ*_I_ = 0.1 and *σ*_E_ = 10.0. The state transitions occur approximately coincidently in individual excitatory neurons, but they can show largely deferent degrees of firing irregularity due to variations in the connection weights (Fig. 3(C,D)). Due to the interconnection between excitatory neurons and inhibitory neurons, inhibitory neurons also show similar bimodal activity patterns (Fig. 3(E)). However, these neurons tend to exhibit more regular membrane potential traces presumably because I-to-I connections are less variable than E-to-E connections.

**Figure 4:**
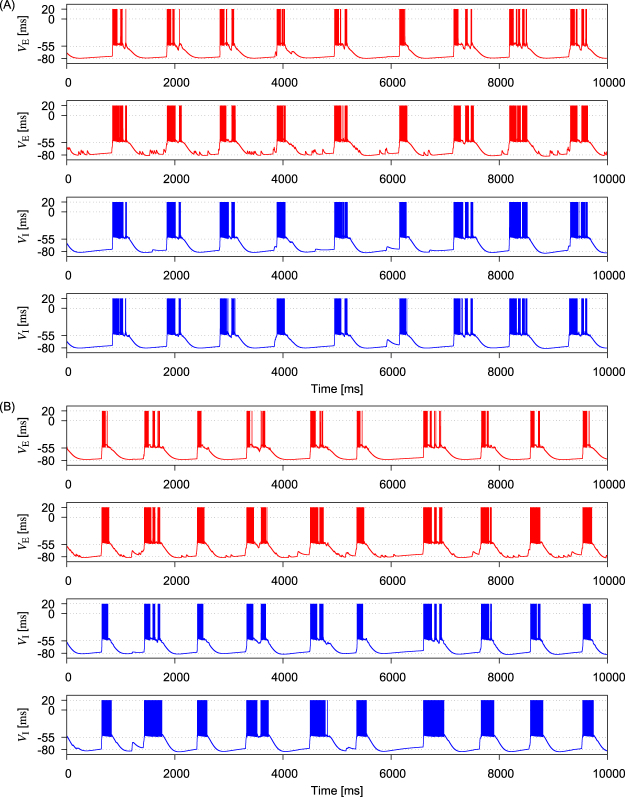
Network activity obtained for sparse-Gaussian and sparse-log-normal weight distributions. (**A**) Membrane potentials of two excitatory neurons and two inhibitory neurons are shown for a sparse-Gaussian weight distribution parameterized as *γ*_E_ = 0.1 and *σ*_E_ = 10. (**B**) Membrane potentials of two excitatory and two inhibitory neurons are displayed for a sparse-log-normal weight distribution with parameters *γ*_E_ = 0.1 and *σ*_E_ = 10.

Networks with sparse-Gaussian (Fig. 4(A)) and sparse-log-normal connection weights (Fig. 4(B)) show similar irregular UP-DOWN transitions, with somewhat less variable temporal activity patterns across neurons compared with patterns for lognorml connection weights. Thus, the irregular temporal patterns of synchronous UP-DOWN transitions represent a feature common to all the network models.

The observations mentioned above raise several questions: (1) What is the condition for UP-state to sustain? (2) How does the duration of UP state or the duty cycle of UP-DOWN transitions vary with the weight configurations of neural networks? (3) How do other statistical features, such as spiking variability during UP states, depend on the weight configurations? (4) What are the possible implications of irregular UP-DOWN transitions in information processing by ensemble neural activity? We will address these questions below.

### Mean-field Analysis

To begin with, we conduct the mean-field analysis of UP states. The purpose of the mean-field analysis is to look for parameters enabling excitatory neurons to give a large enough output firing rate such that excitatory-excitatory interaction is strong enough to support UP states. The central assumption of the mean-field analysis is the homogeneity of the network (or the population). Although homogeneity may not be true in reality, this assumption simplifies the system and allows us to analytically study its properties. In our model, all neurons do not necessarily have exactly the same wiring patterns and connection weights, but connections to different excitatory neurons are statistical the same such that a mean-field analysis is applicable to the network system. Under this assumption, the average firing rates of individual neurons should be identical in the steady state of the neural network although the firing rates of individual neurons fluctuate around the mean. This also implies that the average pre-synaptic firing rate should be identical to the average post-synaptic firing rate because every post-synaptic neuron is pre-synaptic to some other neurons. If this condition is not fulfilled, the network model is unable to reach the corresponding steady state (i.e., the specific UP-DOWN transition state).

**Figure 5:**
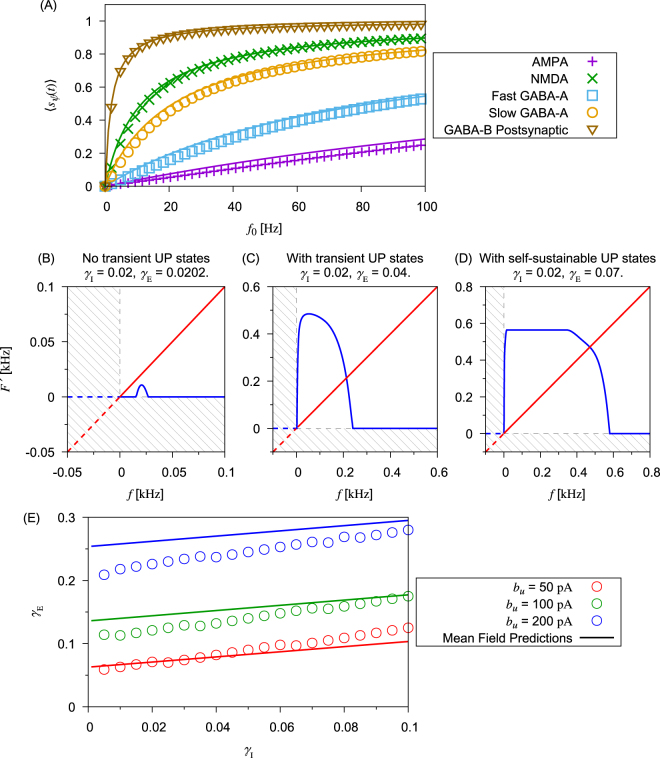
Mean-field analysis of UP-DOWN transition. (**A**) The frequency response of gating functions of different synapses is calculated by numerical simulations of the network models (symbols). Curves represent the predictions by Eq. (14). (**B**–**D**) Schematic illustrations of the mean-field analysis quoted in Eq. (18) with parameters for different scenarios. Blue curves: function *F*′ defined in Eq. (18) as a function of *f*. Red curves: self-consistent constraint. Shaded areas: regions for negative frequencies. (**E**) Comparisons of critical *γ*_E_ for sustainable UP states between predictions by mean-field analysis and simulations for given *γ*_I_’s at different SFA levels.

To study the condition to have UP states, we analyze the behavior of the gating function under the influences of noisy background inputs. By averaging Eqs (3) and (4) over time, we have

14 $${\langle {s}_{\psi ,0}(t)\rangle }_{t}=\frac{{\tau }_{\psi ,{\rm{dn}}}{f}_{0}}{1+{\tau }_{\psi ,{\rm{dn}}}{f}_{0}},$$

where *f*_0_ is the firing rate of the presynaptic neuron. We verified Eq. (14) by numerical simulations (Fig. 5(A)).

Equation (14) allows us to express the temporal average of synaptic current ${I}_{{\rm{syn}}}^{{\rm{X}}}$ as

15 $$\langle {I}_{{\rm{syn}}}^{{\rm{X}}}(t)\rangle \approx {\gamma }_{{\rm{E}}}\sum _{j,\psi }{J}_{\psi ,j}^{{\rm{XE}}}{g}_{\psi }\langle {s}_{j}^{\psi }(t)\rangle ({V}_{{\rm{R}},\psi }-\langle {V}_{{\rm{X}}}(t)\rangle )+{\gamma }_{{\rm{I}}}\sum _{k,\psi }{J}_{\psi ,\,k}^{{\rm{XI}}}{g}_{\psi }\langle {s}_{k}^{\psi }(t)\rangle ({V}_{{\rm{R}},\psi }-\langle {V}_{{\rm{X}}}(t)\rangle ),$$

where the averaging was taken under the assumption that the membrane potential is independent of the gating functions. This assumption simplifies the mean-field calculations though it may not give a true averaging. Assume that during UP states neurons fire at frequency *f*_0_, we obtain

16 $$\sum _{j,\psi }{J}_{\psi ,\,j}^{\mathrm{XE},\mathrm{XI}}{g}_{\psi }\langle {s}_{j}^{\psi }(t)\rangle ({V}_{{\rm{R}},\psi }-\langle {V}_{{\rm{X}}}(t)\rangle )\approx \sum _{\psi }{g}_{\psi }({V}_{{\rm{R}},\psi }-\langle {V}_{{\rm{X}}}(t)\rangle )\frac{{\tau }_{{\rm{dn}},\psi }{f}_{0}}{1+{\tau }_{{\rm{dn}},\psi }{f}_{0}}{N}_{E,I}\langle {J}_{\psi ,j}^{\mathrm{XE},\mathrm{XI}}\rangle $$

Next, we calculate the effect of SFA defined in Eq. (2). By averaging *u*_X_(*t*) with given postsynaptic firing rate *f*_1_, we have

17 $$\langle {u}_{{\rm{X}}}(t)\rangle \approx {a}_{u}(\langle {V}_{{\rm{X}}}(t)\rangle -{V}_{{\rm{L}}})+{b}_{u}{\tau }_{u}{f}_{1}\mathrm{.}$$

As explained previously, in order for neurons to support UP-states, the pre-synaptic firing rate should equal the post-synaptic firing rate, i.e., *f*_1_ = *f*_0_ (≡*f*). Then, from Eqs (10), (15) and (17), we conclude that the system has UP states when the equation

18 $$f=F^{\prime} (f)\equiv F[{I}_{{\rm{syn}}}(f)-{u}_{{\rm{X}}}(f)]$$

has a non-trivial solution to *f*. The existence of the solution to *f* implies that the system allows output from a neuron to balance with its input. Intuitively, balance between the average output and average input of excitatory neurons implies that these neurons support and stabilize mutual excitation among them in their population, as all neurons are simultaneously pre-synaptic and post-synaptic to other neurons. Here *F* is the function defined in Eq. (10). Equation (18) is illustrated in panels (B)–(D) in Fig. 5 for three values of *γ*_E_ and the mean membrane voltage 〈*V*_X_〉 = −55 m*V* during UP states.

The three diagrams shown in panels (B)–(D) in Fig. 5 were used for searching sustainable UP states iteratively. In reality, the values of *γ*_E_ and *γ*_I_ are correlated in the neural network, but here we vary these quantities independently for the illustration purpose. Note that negative frequency is included in the axes to illustrate how the fixed-point solution to Eq. (18) is solved, which makes mathematical sense, but not biological. Same for the range of the axes, it was the range to search for existence of the fixed-point solution only. It does not imply all values showed in the axes are relevant.

In Fig. 5(B), both excitatory activity and inhibition activity are weak, and there is only a trivial fixed point and UP states do not exist. In Fig. 5(C), excitatory activity is larger than that in Fig. 5(B) and there is an additional fixed point, but this state is unstable. Therefore, the network can only sustain a transient UP state for a short period of time. If we further increase excitatory activity, the mean-field analysis gives the diagram shown in Fig. 5(D), which has a non-trivial fixed point corresponding to a self-sustainable UP state. Thus, we can determine the conditions for *γ*_E_ and *γ*_I_ to have transient UP states and self-sustainable UP states.

In Fig. 5(E), the critical value of *γ*_E_ necessary for supporting a self-sustainable UP state was calculated analytically and numerically for given values of *γ*_I_ at different levels of SFA. The analytic results are well consistent with the simulation results for relatively small *b*_*u*_’s. For larger *b*_*u*_, the prediction cannot be so accurate due to the fixed choice of 〈*V*_X_〉. However, the mean-field analysis can still well predict the dependence of *γ*_E_ on both *γ*_I_ and *b*_*u*_.

### Statistics of UP-DOWN Cycles

**Figure 6:**
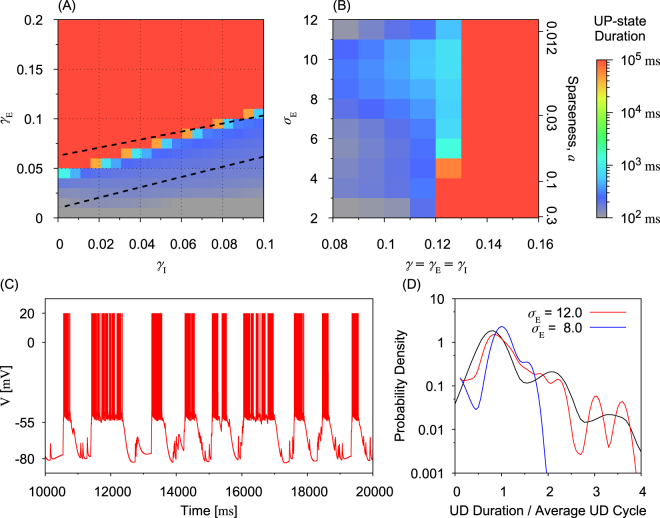
Statistics of UP-DOWN cycle for sparse-log-normal weight configurations. (**A**) The average duration of UP-states is shown for various values of *γ*_E_ and *γ*_I_ at *b*_*u*_ = 50 pA in a sparse-log-normal network of size *N*_E_ = 1000. The upper dashed curve is the predicted boundary from the mean-field analysis for self-sustainable UP states, while the lower dot-dashed curve is the predicted boundary for the presence of the slow oscillations. Sparse-log-normal random connection weights have the fixed variance of ${\sigma }_{{\rm{E}}}^{2}=1$. (**B**) The average duration of UP states at *b*_*u*_ = 200 pA is shown for various values of connection weight factor *γ* and excitatory variance ${\sigma }_{{\rm{E}}}^{2}$ in a network of size *N*_E_ = 1000. (**C**) An example of the membrane trace is shown, which showed a sequence of transient UP states. Parameter values were set as *N*_*E*_ = 1000, *γ* = 0.11 and *σ*_E_ = 12.0. (**D**) Distributions of the UP-state duration of transient UP states normalized by the average UP-state duration are shown for *σ*_E_ = 12.0 (green) and *σ*_E_ = 8.0 (blue). Black curve shows the experimental result reported in^4^. Reprinted by permission from Macmillan Publishers Ltd: Springer Nature. Nature Neuroscience. Spontaneous persistent activity in entorhinal cortex modulates cortico-hippocampal interaction *in vivo*, T. T. Hahn *et al*., copyright (2012). Parameter values were set as *a*_*u*_ = 4.0 nS, *b*_*u*_ = 50.0 nS and *γ* = *γ*_E_ = *γ*_I_ = 0.1.

UP-DOWN transitions can show different characteristics in different cortical areas, presumably reflecting certain differences in the underlying network structure^4^. We therefore compared the stability of UP states and the average duration of repeated UP-states between recurrent networks with the different types of random connection weights. Numerical simulations were conducted for either *N*_*E*_ = 1000 or 2000. In Fig. 6(A), we find that the average duration of UP states is primarily determined by a balance between the average strength of excitatory and inhibitory connections (i.e., by the ratio between the connection weight factors *γ*_E_ and *γ*_I_). The UP states abruptly change from transient ones (deep blue area) to self-sustainable ones (red area) around a boundary specified by a liner function of *γ*_E_ and *γ*_I_. The boundary approximately coincides with the boundary for self-sustainable (almost continuous) UP states predicted by the mean-field analysis. Transient UP states correspond to the slow-oscillation state while self-sustainable UP states are thought to represent the resting state of cortical neurons in awake animals^2^.

We investigated the conditions to have a self-sustainable UP state and found that such a state likely exists if $\gamma (\,=\,{\gamma }_{{\rm{E}}}={\gamma }_{{\rm{I}}})\gtrsim 0.12$ for *N*_E_ = 1000 or if $\gamma (\,=\,{\gamma }_{{\rm{E}}}={\gamma }_{{\rm{I}}})\gtrsim 0.06$ for *N*_E_ = 2000 in all the three connection types. For instance, the former value is given as an intersection between the upper boundary curve and line *γ*_E_ = *γ*_I_ in Fig. 6(A). On the other hand, the average UP-state duration was not very sensitive to weight variance ${\sigma }_{{\rm{E}}}^{2}$ (Fig. 6(B)). We note that since the summations in Eq. (15) scales as *N*_E_, the critical value of connection weight factor scales as 1/*N*_E_ in our mean-field analysis (see the previous sub-section). A similar scaling rule applies to the variance ${\sigma }_{{\rm{E}}}^{2}$, which scales with $\sqrt{{N}_{{\rm{E}}}}$. In Fig. 6(B), the boundary of sustainable UP states ($\gamma \gtrsim \mathrm{0.12)}$ predicted by the mean-field theory is valid for *σ*_E_ < 12 when *N*_E_ = 1000, but the mean-field theory only works for *σ*_E_ < 14 when *N*_E_ = 2000 (see Supplementary Fig. S3(C)). Other network models with log-normal random connection weights (see Supplementary Fig. S1) and sparse-Gaussian random connection weights (see Supplementary Fig. S2) also show similar phase diagrams. Thus, the detailed statistical properties of connection weights did not strongly influence the macroscopic network dynamics studied here.

It has been shown that the layer 3 of the medial entorhinal cortex (MECIII) shows prolonged UP states that can persist up to several cycles of slow-wave oscillations, whereas in the layer 3 of the lateral entorhinal cortex (LECIII) UP states only persist within single oscillation cycles^4^. Thus, different cortical areas can exhibit significantly different temporal profiles of UP states, presumably reflecting certain differences in the underlying network structure. We therefore investigated how the temporal profiles of UP-DOWN transitions vary with the choice of connection weights. When the connection weight variance ${\sigma }_{{\rm{E}}}^{2}$ is sufficiently large, UP-DOWN transitions in our models display highly irregular temporal profiles resembling persistent UP states in MECIII (Fig. 6(C)). Accordingly, in all the weight distributions, the distribution of UP-DOWN-cycle duration exhibits multiple peaks for relatively large values of *σ*_E_ whereas the distribution only has a single peak, as in LECIII, for smaller values of *σ*_E_ (Fig. 6(D) and Supplementary Fig. S4). In these simulations, the durations of UP-DOWN-cycle were presented in the unit of the average UP-DOWN cycle duration for given set of parameter values. The definition of UP-DOWN cycle duration was the sum of durations of an UP state and a consecutive DOWN state. The probability density function is the derivative of the cumulative distribution of UP-DOWN cycle durations of all excitatory neurons in the network. We found that the connection weight factor did not significantly modulate variability in the temporal profiles of UP-DOWN transitions. Thus, our results suggest that the variance in connection weights, but not their average, influences the temporal variability of UP-DOWN transitions.

It is noticed that the UP-DOWN-cycle durations were clearly discretized in experiment, while such a tendency was only poorly expressed in our simulations for all the connection weight configurations (Fig. 6(D) and Supplementary Fig. S4). The reason for this discrepancy between the models and experiments remains unclear. The discrepancy might be due to a slight difference in the definition of normalized UP-DOWN-cycle duration between the models and experiment, in which the UP-DOWN-cycle duration was normalized by the average duration of UP-DOWN cycles in the neocortex^4^. However, this is unlikely because the difference in the unit time would not eliminate the discretized nature of the distribution. Rather, the discrepancy may reflect certain influences of external input to the local cortical circuits, which was simply modeled as Poisson spike trains in the present study whereas the UP-DOWN transitions in MECIII were correlated with those in other cortical networks (LECIII and neocortex). Alternatively, the discrepancy may be due to the complexity of local cortical circuits that was also not modeled here. In this work, the causes of the discrepancy will not be further explored.

### Statistics of Irregular Neuronal Firing

**Figure 7:**
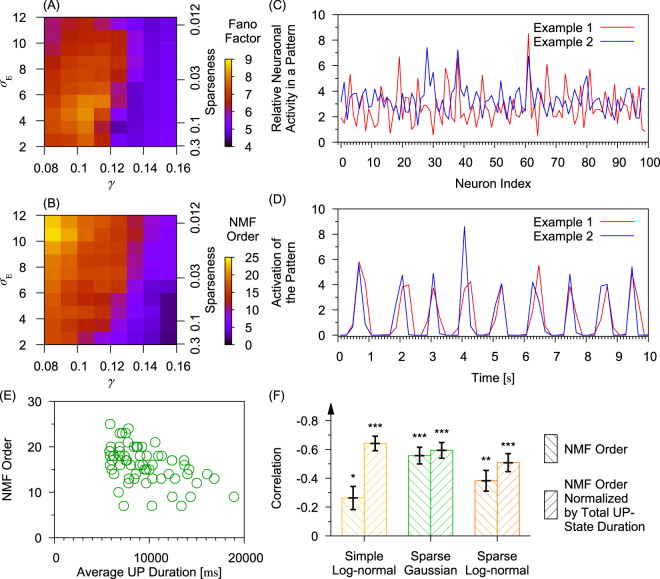
Cell-assembly structure of UP states in sparse-lognomal networks. (**A**) Fano factors measured at different parameter values for sparse-log-normal connection weight distributions. Parameter: *c*. (**B**) Optimal NMF order detected by AICc within the same parameter range used in (**A**). (**C**) Two examples of the ensemble activity patterns during transient UP states detected by NMF are displayed. The firing rates are only shown for 100 neurons selected randomly from the network. (**D)** Occurrence of the two patterns extracted by NMF is shown during repeated UP states. Parameters for (**C** and **D**): *N*_E_ = 1000, *γ*_I_ = *γ*_E_ = 0.1 and *σ*_E_ = 5.0. (**E**) The optimal number of patterns is plotted against the average transient-UP-state duration. (**F**) Correlations were calculated between the original and normalized NMF orders and logarithms of the average transient-UP-state duration. Asterisks: *p*-values for the significant levels of the negative correlation coefficients. *p < 0.05. **p < 0.01. ***p < 0.001.

Spike trains during UP States are highly variable in all the three networks. To see this, we calculated Fano factors of spike trains during UP-states repeated in each network as a function of *γ* and *σ*_*E*_ (Fig. 7(A)), where Fano factor is defined as the variance of spike counts over the repetition divided by the average spike count. Fano factor was introduced by a physicist^31^. Fano factor is now widely used in Neuroscience as a measure for spike-timing variability. Here, the Fano factor is calculated on a set of spikes in which inter-spike intervals overlapping with DOWN states are eliminated. We calculated Fano factors in the region of parameter space in which UP states occur. The Fano factors presented are the average of all excitatory neurons in the network.

In simulations, we found that Fano factor depends primarily on the connection weight factors and abruptly becomes small as the value of *γ* is increased. The critical value of *γ* coincides with the boundary for sustainable UP states predicted by the mean-field analysis. In contrast, Fano factor does not significantly depend on the weight variance. Interestingly, the regions in the parameter space giving large Fano factors mostly exclude the regions for larger UP-state durations (c.f. Fig. 6(B)). This implies that spike trains are more variable during repeated UP states when these states have shorter average duration. We note that the different types of random connection weights show similar results (see Supplementary Figs S5(A) and S6(A)). Below, we examined whether this result has implications for cortical memory processing during the slow-wave sleep.

### Repetition of Rate-Coding Neural Ensembles during UP states

We found that the recurrent networks activate a set of cell assemblies during UP states, where a cell assembly is defined as a group of neurons that are synchronously active in a certain time window. As cell assemblies are thought to play a pivotal role in memory processing, we analyzed the instantaneous patterns of simultaneously activate neurons. Sampling the firing rates of neurons at 20 Hz, we calculated the time series of population rate vectors over non-overlapping sliding time windows of 200 ms. The vectors of population firing rates throughout the simulation formed a data matrix **D** of which columns are the instantaneous firing rates of all excitatory neurons at different times and rows represent the time series of firing rates of single neurons. A data clustering technique namely non-negative matrix factorization (NMF) is applied to search for best approximated matrix product to approximate the data matrix, i.e. **D** ≈ **BC**^32^. This technique is suggested to be a generalized *k*-means method^33^. The order of the factorization, i.e. number of columns of matrix **B**, is determined by Akaike’s information criterion with second order adjustment (AICc)^34^. The separation chosen by AICc is the result with least information loss. The orders of NMF for different combinations of parameters are shown in Fig. 7(B) for sparse-log-normal random connection weights. Similar results are shown for log-normal random connection weights and sparse-Gaussian random connection weights in Supplementary Figs S5(B) and S6(B), respectively. Two examples of column vectors of matrix **B** are plotted in Fig. 7(C), which can be interpreted as a co-activated firing pattern detected by NMF. The corresponding row vectors in matrix **C** are shown in Fig. 7(D), which can be interpreted as the activation of the pattern.

The NMF order is negatively correlated with the average UP-state duration in all the three networks (Fig. 7(E) and Supplementary Fig. S7), as verified quantitatively in Fig. 7(F) together with the NMF order normalized by the total UP-state duration during multiple UD cycles. Here the correlations are calculated base on the data point shown in Figs 7(E), and S7 respectively. These results imply that neural population exhibits the repertoire of activity patterns mostly in the initial phase of UP states (within a few hundred milliseconds from the onset of UP states). In other words, a prolonged UP state repeatedly activates a similar set of cell assemblies without adding novel activity patterns with the time passage. This result is reasonable because the repertoire of activity patterns is primarily determined by the spatial configuration of synaptic connections, which remains unchanged during the individual UP states. Further, the repetition of similar cell assemblies is likely to be necessary for long-term memory consolidation, which is thought to occur during slow-wave oscillation.

**Figure 8:**
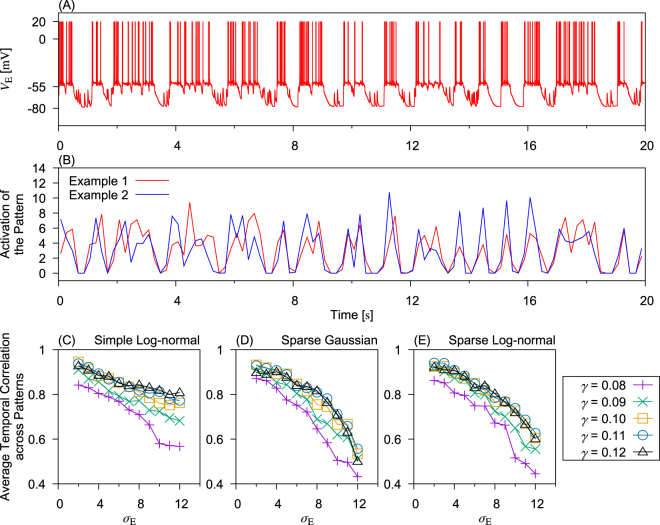
Relation between pattern activation correlation and *σ*_E_. (**A**) Membrane potential of an excitatory neuron is shown in a sparse-log-normal network. The parameters were set as: *N*_E_ = 1000, *γ* = 0.12 and *σ*_E_ = 12.0. (**B**) Activation trace of two example NMF-extracted patterns are presented for the simulation shown in panel (A). (**C**–**E**) Average correlations across NMF-extracted patterns are shown in random networks with log-normal, sparse-Gaussian and sparse-lognormal connection weights. The network size *N*_E_ = 1000.

We further analyzed whether different temporal profiels of UP-DOWN cycles have any implication for the activation of multiple cell assemblies. In the sparse-lognormal network shown in Fig. 7(D), the activation traces of two example cell-assembly patterns are highly correlated and their peak activation times within each UP state are only slightly different. However, the magnitude of *σ*_E_ can affect the coactivation patterns of multiple cell assemblies as it extensively modulates the UP-DOWN cycle (see Fig. 6(D) and Supplementary Fig. S4). We therefore increased the value of *σ*_E_ to obtain irregular UP-DOWN cycles with more-frequent prolonged UP states (Fig. 8(A)). Interestingly, the activation traces of two NMF-extracted cell-assembly patterns are less correlated compared to the previous case, especially during prolonged UP states (Fig. 8(B)). Results for different network configurations are summarized in Fig. 8(C–E), in which the average temporal correlation across different pairs of patterns is negatively correlated with *σ*_E_. Since larger *σ*_E_ on average results in longer UP states (Fig. 6(D)), these results show a negative correlation between UP-state duraion and coherence among cell assemblies are during prolonged UP states. Because the decreased temporal coherence resulted from the increased alteration of cell assemblies, prolonged UP states imply an enhanced alteration among cell assemblies. This network behavior predicted by our model may be of functonal importance for binding together the activities of neurons for cortical memory processing.

## Discussion

### Model-independent Features of Macroscopic Network Behavior

In this study, a family of networks of spiking neurons were used to investigate the dependence of slow-oscillatory network behavior on the distributions of random connection weights. A neuronal model based on adaptive exponential integrate-and-fire model^18^ with a finite refractory period was employed. Compared to the well-known leaky-integrate-and-fire model, this model is complex enough to generate temporal profiles of the membrane potentials comparable to those of cortical neurons^2,3,35^. Nevertheless, this model is less complicated than the Hodgkin– Huxley model^36^, for which simulations of a large-scale network would consume too much computational power. In addition, the neuron model used in this study does not have bi-stability, allowing us to focus on the properties of neural dynamics originating from various network configurations.

In comparison with the previous studies on networks of neurons connected with sparse recurrent connections^13,15^, this study considered a broader class of connectivity. Three classes of random connections were considered, that is, fully-connected log-normal random connection weights, sparse-Gaussian random connection weights and sparse-log-normal random connection weights. Although the fully-connected neural network is unrealistic, it was studied for comparison to sparse neural networks. For fair comparisons, we chose the parameter values of different distributions such that their means and variances took approximately the same values.

While the mean strength of connections is crucial for the occurrence of UP states and variability in spike trains (Fano factor) during the UP states, the variance of weight distributions is influential on the existence and variety of persistent UP states (Fig. 6(D)). In contrast, our results suggest in a wide range of parameter values that neither the detailed profiles of weight distributions nor the higher moments of these distributions are important for any macroscopic network behavior studied here (Fig. 6(B), and Supplementary Figs S1(B) and S2(B)). For example, in Fig. 6(B) the critical connection strength *γ* takes similar values in a wide value range of the variance, which agrees with the prediction of the mean-field analysis, as verified in Fig. 6(A) and illustrated in Fig. 5(B–D). This result suggests that the network configuration can modulate the temporal patterns of the UP-DOWN transition, but only the average strength of connections is important for the emergence of UP-DOWN transition. Nevertheless, the propagation of spatiotemporal spiking patterns during UP-state transitions is potentially model-dependent due to different connection configurations. However, such an analysis was not attempted in this study because it requires a methodology that offers a higher temporal resolution than the NMF method, which was applied to the spatiotemporal patterns of firing rate.

### Persistent UP States in Network Models and MECIII

As shown in Figs 3 and 4, our model generates the membrane potential traces comparable to those observed in experiments^2–4,35^. Driven by Poisson-spike trains, the membrane potentials of model neurons oscillate at an average frequency of ~$1\,{\rm{H}}z$, as in experiment. However, there is some discrepancy between experiments and simulations in the statistics of UP-DOWN cycles (Fig. 6(D)). Experimental results from MECIII shows a clear multimodal trend in the cumulative distribution of UP-DOWN cycles, but such a tendency is less obvious in the results of our modeling study.

The actual cause of this discrepancy remains unclear. However, a possible cause is that the MECIII network interacts with LECIII and neocortical areas to receive oscillatory driving inputs. This could be speculated from correlations in slow oscillatory activity between MECIII and these areas^4^. In this case, the onsets of UP-DOWN cycles are expected to be phase-locked among these areas. However, because our network models did not receive such input and were merely driven by Poisson-spike trains, the cumulative curves would not show clear multimodal trends.

Our analysis based on NMF of neural population activity suggest that each UP state repeats an approximately identical set of ensemble activity patterns without increasing the repertoire of patterns (Fig. 7(E)). This result is interesting because it implies that MECIII may consolidate long-term memory more robustly than LECIII by utilizing prolonged UP states. Especially, our results suggest that the activation of different cell-assembly patterns is less temporally correlated and the dominant patterns switch more often in such networks as support longer UP states (Fig. 8). This result implies that the cortical region MECIII, which shows prolonged UP states, may retrieve cell-assembly patterns in a manner different from that of LECIII. In reality, however, ensemble activity patterns may slowly vary through synaptic plasticity during the repetition of UP states, so the above prediction of the models should be interpreted with some limitation. This result suggests that network configurations affects the occurrence probability and behaviors of slow oscillations, which will further affect information processing during slow oscillations. These functional implications of persistent UP states should be further clarified experimentally and computationally.

### Validity of Mean-field Approach

Mean-field analysis was developed as an approach in the statistical mechanics to spin systems such as Ising models^37^, and has been applied to various random neural network models^38,39^. A crucial assumption of the mean-field analysis is that the network structure should be homogeneous. Because the connectivity used in the current models is random and uniform, a mean-field approach should be sufficient for studying their steady states. So, the mean-field analysis enabled us to derive the stability conditions for active network states in terms of the average firing rate of neurons (Fig. 5(B–D)). However, these results are not necessarily valid for the stability of random neural networks of spiking neurons because such networks generally have dynamical states for which a rate description is inaccurate (e.g., synchronously firing states). For example, in Fig. 5(D), if the fluctuation of instantaneous firing rate is very large, the firing rate of neurons can easily drop to a small value and the UP state cannot be sustained for a long time, implying that the UP state may not be “self-sustainable”. Nevertheless, the actual discrepancy between the analytical and numerical results is not significantly large in the present study (Fig. 5(E)).

One may query whether the mean-field analysis require all neurons in the brain to fire at the same firing rate. In the calculation, the synaptic current is effectively a sample sum of neurons with different firing rate. Also, the synaptic current used in Eq. (18) is also an average among all neurons. So the mean-field analysis looks for conditions that average output firing rate matches average input firing rate to make UP-state transition possible. Further, since neurons are mainly connected with its neighboring neurons in the same column, the condition we obtained in this study is mostly a local condition rather than a global condition in the brain.

An interesting implication of our mean-field analysis is that the conditions for the neuronal network to support self-sustained UP states and transient UP states are mostly independent of the types of random connection weights. In simulations, we found that the mean-field analysis well predicted the macroscopic network behavior in a considerably large range of the variance of random connection weights. Not only the conditions for the sustainability of UP-states, but also the UP-state duration (Fig. 6, and Supplementary Figs S1 and S2) and Fano factors of spike trains during the UP states (Fig. 6(E), and Supplementary Figs S4(A) and S5(A)) are qualitatively independent of the types of random connection weights. Although these results are yet to be confirmed in larger neural networks, the results were qualitatively unchanged when the network size was doubled in the present study.

### Relations to previous modeling studies

A network model of spiking neurons was previously proposed to address the biological mechanism of two-state membrane potential fluctuations^13^. The authors used biologically realistic conductance-based neuron models to study the neural mechanisms to maintain excitation-inhibition balance during slow oscillation^40,41^ and the effects of pharmacological manipulations on collective network behavior. For these purposes the choice of realistic neuron models is essential. In contrast, our model consists of adaptive exponential leaky integrate-and-fire neurons^18^, which are not realistic enough to study the effects of pharmacological manipulations. However, this neuron model is simple enough for mathematically analyzing the influences of the E-I ratio, strength of SFA, critical value of *γ* and weight variance on the self-sustainability of UP states. In biological systems, some of these parameters may change according to the brain state. For example, changes in extracellular chemical concentrations such as calcium^42–44^ can change the firing patterns of neurons, which may in turn changes the effective E-I ratio in cortical networks. The present results enrich our knowledge on which network configurations may or may not affect the characteristics of UP states and UP-DOWN transition.

A network model consisting of plastic synapses and spiking neurons was constructed to examine the activity-dependent self-organization and maintenance of excitation-inhibition balance^15^. The model suggested that some excitatory synapses are selectively strengthened but the remaining majority are down-scaled during the repetition of UP states. Synaptic downscaling or synaptic homeostasis has been hypothesized as the role of NREM sleep in learning^45^. However, accumulating evidence suggests that NREM sleep promotes the selective strengthening of synapses and the creation of new synapses in an experience-dependent manner^46–48^. Refinement of synaptic connections and its effects on slow-wave oscillatory activity were also recently studied in a large-scale model of visual thalamocortical circuits^49^. Although the present model does not include modifiable synapses and hence tells nothing about these issues, some of the predictions, in particular the recurrence of similar ensemble activity patterns during each UP state, has implications for the understanding of effects of NREM sleep on memory consolidation.

Local cortical circuits are known to be highly non-random^50–54^. A computational model suggested that clustered synaptic connections make the temporal profile of slow oscillatory activity highly variable^16^. Our results propose a simpler account for such a variability when the variance of synaptic weight distributions is sufficiently large. In such a case, different levels of variability can be obtained for different levels of the variance of random connection weights (Fig. 6(D)). However, the two mechanisms are not mutually exclusive, and both of them in reality may contribute to the recurrence of highly variable temporal patterns during slow oscillatory activity. It is intriguing to study the bistable network behavior when synaptic connections with a large weight variance have a clustering structure^55^.

### Biological implications of our findings

In this paper, we presented numerical and analytic results on the dynamics of UP states and UP-DOWN transitions in randomly-connected recurrent neural networks with various distributions of connection weights. Conditions for generating transient UP states and self-sustainable UP states were analytically derived and confirmed by numerical simulations. These results provided a comprehensive picture of the dynamical phenomena regulated by excitatory connections, inhibitory connection, spike-frequency adaptation and slow oscillatory activity. The emergence of UP states, at least in the current model, does not qualitatively depend on the higher-order statistical features of neuronal wiring. Our results suggest that UP-DOWN transitions occur robustly for a wide range of weight configuration, as far as the mean and variance of connection weight distributions are given in adequate ranges.

Some network configurations give highly irregular UP-DOWN transition cycles together with prolonged UP-state durations. While the temporal profiles of UP-DOWN cycles are insensitive to variations in weight distribuion, a large variance of connection weights may produce irregular UP-DOWN cycles similar to those reported in Hahn *et al*.^4^. Our model suggests that the emergence of continous UP-states in MECIII, but not in LECIII, is due to different variances of connection weights in the two regions. Further, our model suggests that cell assemblies are evoked differently during UP states in networks with different variances of connection weights. Evoked cell-assembly patterns change more frequently in networks with larger variances of connection weights. All together, our results suggest that cell-assembly dynamics of MECIII and LECIII are different during memory consolidation.

## Electronic supplementary material

Supplementary Information
