# Supplementary material for: Transient and Persistent UP States during Slow-wave Oscillation and their Implications for Cell-Assembly Dynamics
Source: Sci Rep. 2018 Jul 16;8:10680. doi: 10.1038/s41598-018-28973-y (PMC6048140; doi:10.1038/s41598-018-28973-y)
Supplement: Supplementary file 1 — Supplementary Information [file 41598_2018_28973_MOESM1_ESM.pdf]

Supplementary information for the article "Transient and Persistent UP States during Slow-wave Oscillation and their Implications for Cell-Assembly Dynamics"

Chi Chung Alan Fung and Tomoki Fukai

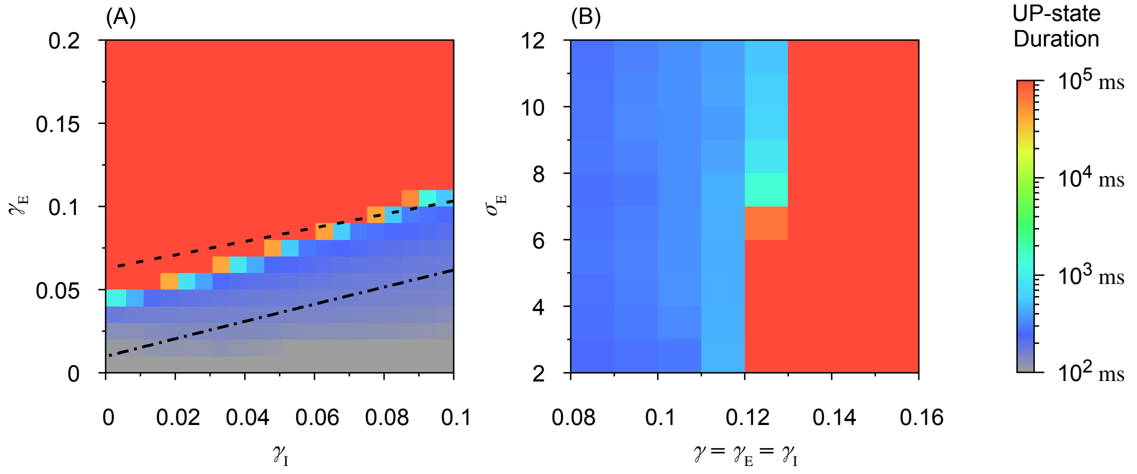

Fig. S1: Additional plots for panels (A) and (B) of Fig. 6 for log-normal random couplings. (A) Average duration of UP states in the parameter space spanned by γ_E and γ_I. (B) Average duration of UP states in the parameter space spanned by γ and σ_E. Parameters: same as Fig. 6.

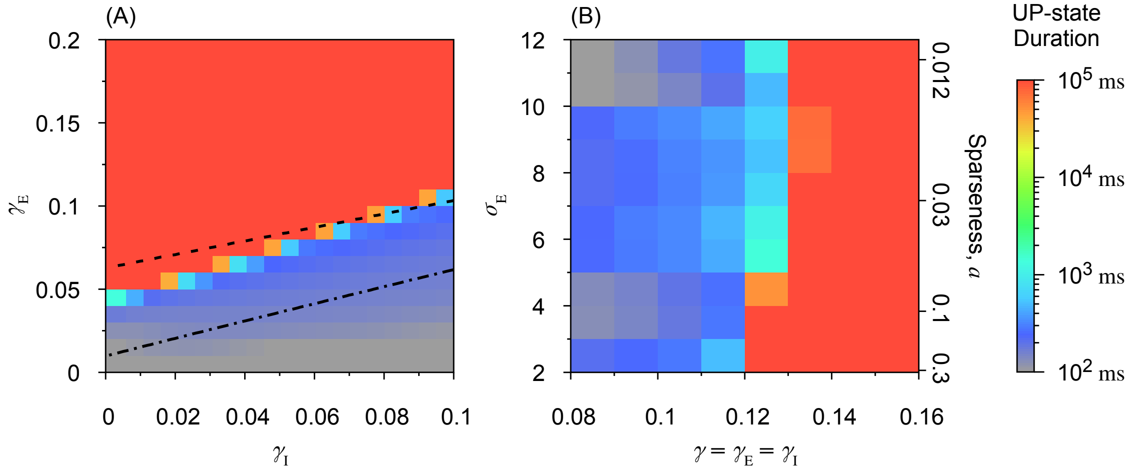

Fig. S2: Additional plots for panels (A) and (B) of Fig. 6 for sparse-Gaussian random couplings. (A) Average duration of UP states in the parameter space spanned by γ_E and γ_I. (B) Average duration of UP states in the parameter space spanned by γ and σ_E. Parameters: same as Fig. 6.

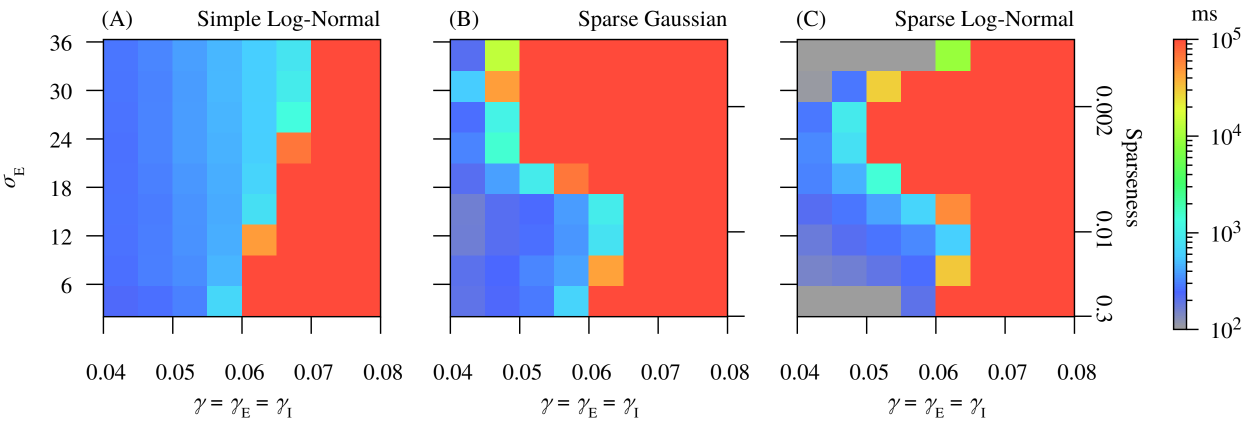

Fig. S3: Additional plots for panel (B) of Fig. 6 for $N = 2000$. Average duration of UP states in the parameter space spanned by γ and σ_E. (A) Simulations with log-normal random couplings. (B) Simulations with sparse-Gaussian random couplings. (C) Simulations with sparse-log-normal random couplings.

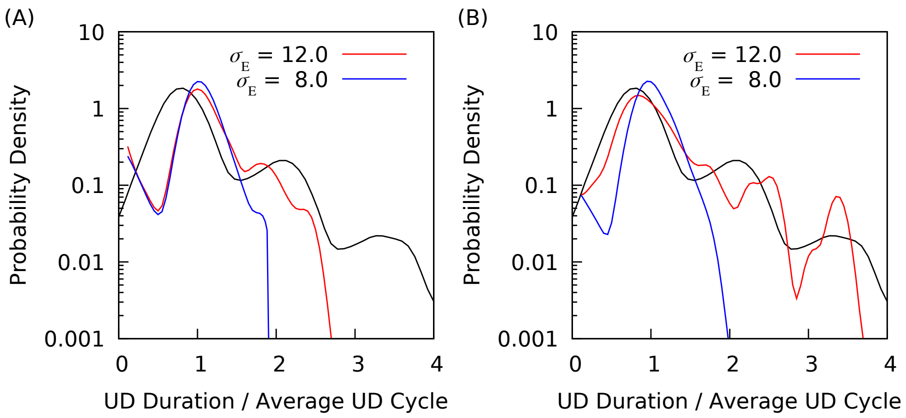

Fig. S4: Distributions of UP-DOWN cycles. (A) Distribution for log-normal random couplings. (B) Distribution for sparse-Gaussian random couplings. Black solid lines: experimental results by Hahn et al in 2012. Reprinted by permission from Macmillan Publishers Ltd: Springer Nature. Nature Neuroscience. Spontaneous persistent activity in entorhinal cortex modulates cortico-hippocampal interaction *in vivo*, T. T. Hahn *et al.*, copyright (2012). Color solid lines: simulation result. Parameters: same as Fig. 6.

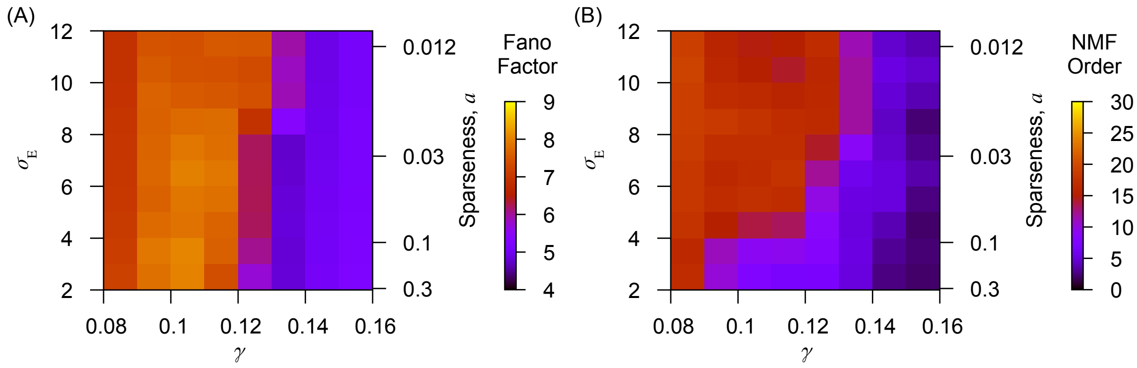

Fig. S5: Fano factor and NMF order for log-normal random couplings. (A) Fano factor of spikes during UP states in a neurons network with log-normal random couplings. (B) NMF Order of firing rate in a neurons network with log-normal random couplings. Parameter values are the same as in panel (A) and (B) in Fig. 7.

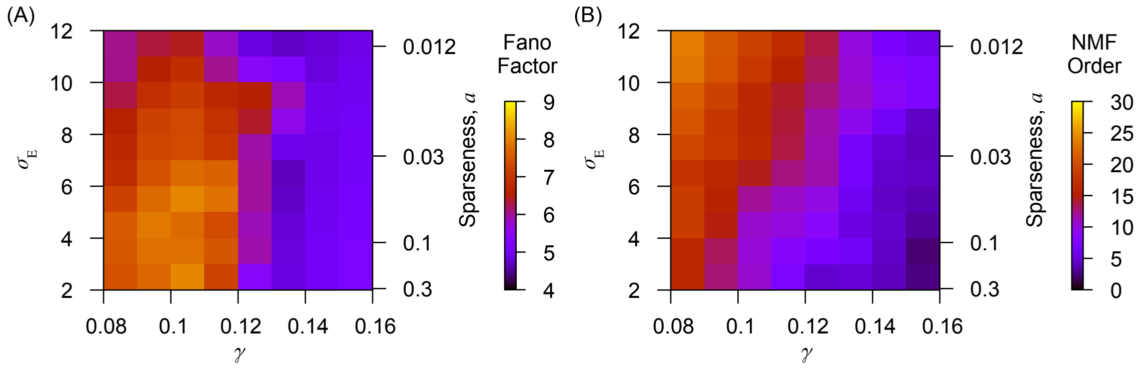

Fig. S6: Fano factor and NMF order for sparse-Gaussian random couplings. (A) Fano factor of spikes during UP states in a neurons network with sparse-Gaussian random couplings. (B) NMF Order of firing rate in a neurons network with sparse-Gaussian random couplings. Parameter values are the same as in panels (A) and (B) in Fig. 7.

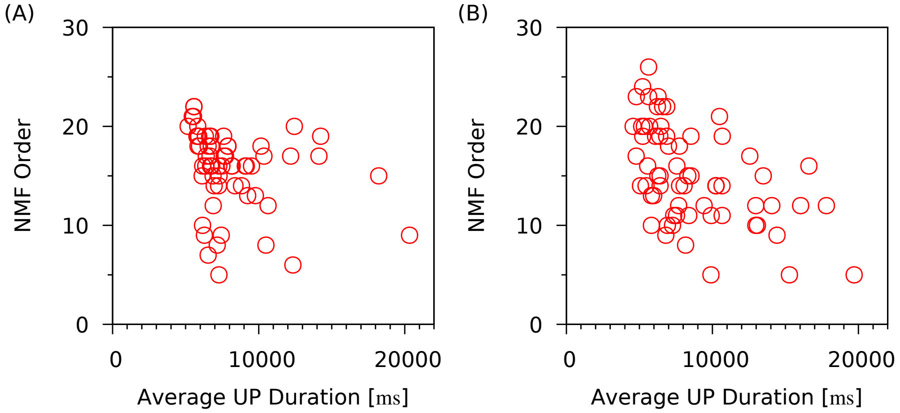

Fig. S7: NMF order versus average UP-state durations in various simulations. (A) Simulations in neuronal networks with log-normal random couplings. (B) Simulations in neuronal networks with sparse-Gaussian random couplings. Parameters values are the same as in panel (E) in Fig. 7.
